# Mortality and readmission rates among hospitalized COVID-19 patients with varying stages of chronic kidney disease: a multicenter retrospective cohort

**DOI:** 10.1038/s41598-022-06276-7

**Published:** 2022-02-10

**Authors:** Brent Appelman, Jetta J. Oppelaar, Lani Broeders, Willem Joost Wiersinga, Hessel Peters-Sengers, Liffert Vogt, the CovidPredict Study Group, Brent Appelman, Michiel Schinkel, David Buis, Kim C. E. Sigaloff, Paul W. G. Elbers, Daisy Rusch, Auke Reidinga, Hazra Moeniralam, Caroline Wyers, Joop van den Bergh, Suat Simsek, Bastiaan van Dam, Niels C. van den Gritters, Nejma Bokhizzou, Kees Brinkman, Martijn de Kruif, Tom Dormans, Renée Douma, Lianne R. de Haan, Tsz Yeung Fung, Martijn Beudel

**Affiliations:** 1 Center for Experimental and Molecular Medicine, Amsterdam UMC, Amsterdam, The Netherlands; 2 The Amsterdam Institute for Infection and Immunity, Amsterdam UMC, Amsterdam, The Netherlands; 3 Department of Internal Medicine, Section of Nephrology, Amsterdam UMC, Amsterdam, The Netherlands; 4 Division of Infectious Diseases, Location Academic Medical Center, University of Amsterdam, Amsterdam UMC, Amsterdam, The Netherlands; 5 Department of Intensive Care, Amsterdam UMC, Amsterdam, The Netherlands; 6 Department of General Practice, Martini Hospital, Groningen, The Netherlands; 7 Intensive Care Unit, Martini Hospital, Groningen, The Netherlands; 8 Department of Internal Medicine and Intensive Care Unit, St Antonius Hospital, Nieuwegein, The Netherlands; 9 Department of Internal Medicine, Viecuri MC Noord-Limburg, Venlo, The Netherlands; 10 Department of Internal Medicine, Noordwest Ziekenhuiszgroep, Alkmaar, The Netherlands; 11 Intensive Care Unit, Treant Zorggroep, Emmen, The Netherlands; 12 Department of Internal Medicine, BovenIJ Hospital, Amsterdam, The Netherlands; 13 Department of Internal Medicine, OLVG, Amsterdam, The Netherlands; 14 Department of Pulmonary Medicine, Zuyderland Medical Centre Heerlen, Heerlen, The Netherlands; 15 Department of Internal Medicine, Zuyderland Medical Centre Heerlen, Heerlen, The Netherlands; 16 Department of Internal Medicine, Flevoziekenhuis, Almere, The Netherlands; 17 Department of Internal Medicine, Section of Nephrology, Maastricht UMC, Maastricht, The Netherlands; 18 Department of Neurology, Amsterdam Neuroscience Institute, Amsterdam UMC, Amsterdam, The Netherlands

**Keywords:** Infection, Infectious diseases

## Abstract

Chronic kidney disease (CKD) has been recognized as a highly prevalent risk factor for both the severity of coronavirus disease 2019 (COVID-19) and COVID-19 associated adverse outcomes. In this multicenter observational cohort study, we aim to determine mortality and readmission rates of patients hospitalized for COVID-19 across varying CKD stages. We performed a multicenter cohort study among COVID-19 patients included in the Dutch COVIDPredict cohort. The cohort consists of hospitalized patients from March 2020 until July 2021 with PCR-confirmed SARS-CoV-2 infection or a highly suspected CT scan-based infection with a CORADS score ≥ 4. A total of 4151 hospitalized COVID-19 patients were included of who 389 had a history of CKD before admission. After adjusting for all confounding covariables, in patients with CKD stage 3a, stage 3b, stage 4 and patients with KTX (kidney transplantation), odds ratios of death and readmission compared to patients without CKD ranged from 1.96 to 8.94. We demonstrate an evident increased 12-week mortality and readmission rate in patients with chronic kidney disease. Besides justified concerns for kidney transplant patients, clinicians should also be aware of more severe COVID-19 outcomes and increased vulnerability in CKD patients.

## Introduction

Chronic kidney disease (CKD) has been recognized as a highly prevalent risk factor for both the severity of coronavirus disease 2019 (COVID-19) and COVID-19 associated adverse outcomes^1,2^. Mortality rates of COVID-19 attributed to CKD may vary between 1.3% and 21.3%^3,4^. Whether disease severity and mortality rates depend on the CKD stage or are explained either by a higher prevalence of comorbid conditions in COVID-19 or by kidney-specific factors, such as uremic or immunological factors, is incompletely known. In January 2021, the European Renal Association and European Dialysis and Transplantation Association (ERA-EDTA) called for action to include all CKD stages in COVID-19 related clinical research^5^. So far, most reports about COVID-19 severity and outcomes do not include all CKD stages and direct comparisons with non-CKD patients are limited^3,4^. Also, a complete characterization of CKD patients hospitalized for COVID-19, including clinical and laboratory data at hospital admission, is infrequently reported. In this multicenter observational cohort study, we aim to determine mortality and readmission rates of patients hospitalized for COVID-19 across varying CKD stages.

## Materials and methods

### Study population and definitions

We performed a multicenter cohort study among COVID-19 patients included in the Dutch COVIDPredict cohort^6–8^. The COVIDPredict is a consortium of eleven hospitals in the Netherlands that aim to understand better and predict which COVID-19 patients should receive which treatments and which type of care. All methods were carried out in accordance with relevant guidelines and regulations. The study protocol was reviewed by the medical ethics committees of the Amsterdam University Medical Centers (Amsterdam UMC; 20.131). The need for informed consent was waived by the Institutional Review Board of Amsterdam University Medical Centres. An opt-out procedure was communicated by press release according to national guidelines and the European privacy law.

The cohort consists of hospitalized patients from March 2020 until July 2021 with PCR-confirmed SARS-CoV-2 infection or a highly suspected CT scan-based infection with a CORADS score ≥ 4^9^. Patients that were transferred from another hospital with an initial admission date > 48 h, readmission records and CKD patients with an undefined CKD stage pre-admission were excluded. Seven groups of CKD were defined based on the previously reported medical history of CKD. The pre-admission kidney condition was calculated with CKD-EPI formula^10^ and the eGFR was used to categorized CKD-stages according to the Kidney Disease Improving Outcomes stages of CKD stages^11^. Patients without CKD were used as controls (“no-CKD”).

### Outcomes and statistical analyses

We compared comorbid risk factors, baseline vitals and laboratory values, and disease severity across groups. Parametric variables were presented as mean and standard deviation (SD); for non-parametric data, the median and interquartile range (IQR) was used. Dichotomous data were presented with frequencies (n) and percentages (%). To compare differences for continuous data, we used a one-way ANOVA or Kruskal–Wallis (KW) test, depending on the type of distribution. If the overall group was deemed significant, posthoc pairwise t-tests (after one-way ANOVA) or Wilcoxon rank tests (after KW) were performed (Benjamini-Hochberg (BH), adjusted) to compare no CKD to the CKD group. For categorical data, we used the Fisher exact test to compare group differences, with pairwise posthoc tests (BH adjusted) with the no CKD group if overall significant. The primary outcome was a composite endpoint of all-cause mortality, readmission or palliative hospital discharge in a 12-week follow-up period. Logistic regression was used to adjust the association between CKD groups and primary outcome in a stepwise procedure for the following confounders: age, sex, ethnicity, number of comorbidities, and designated COVID-19 treatment wave, reflective of the introduction of dexamethasone as a standard treatment regimen^12^. Overall, < 2% observations were missing, which were entirely considered at random and listwise omitted from the adjusted analyses. A *P* ≤ 0.05 was considered significant. Data analyses were performed with R (v3.6.1)^13^.

## Results

### Risk factors and COVID-19 severity

Of the 5153 hospitalized COVID-19 patients enrolled, 755 were excluded because the admission was a readmission of COVID-19, it concerned a transfer from another hospital or were lossed to follow-up. A total of 247 patients with CKD were excluded due to missing pre-admission creatinine values and incomplete CKD stage information (Supplementary Fig. 1). In the remaining 4151 patients, 389 (9.4%) patients were labeled as CKD patients and 3762 (90.6%) patients as no-CKD patients. At admission, baseline characteristics between groups were significantly different for age, ethnicity, designated treatment wave and prevalence of registered comorbidities (Table 1). There were no significant differences in disease severity on admission as measured with MEWS, qSOFA and CRP levels (Tables 1, 2).

**Table 1:** Demographics, comorbidities and outcomes in patients hospitalized for COVID-19 among chronic kidney disease groups.

	No CKD	CKD stage 2	CKD stage 3a	CKD stage 3b	CKD stage 4	CKD stage 5	Dialysis	Kidney transplantation	P value
	n = 3762	n = 50	n = 75	n = 94	n = 49	n = 21	n = 43	n = 57	
**Demographics**									
Sex = Male (%)	2280 (60.7)	36 (72.0)	48 (64.0)	56 (59.6)	29 (59.2)	14 (66.7)	24 (55.8)	30 (52.6)	0.609
Age (median [IQR])	65.00 [55.00, 75.10]	71.00 [65.00, 77.53]*	76.00 [69.50, 82.00]*	77.00 [70.00, 82.75]*	78.10 [71.00, 82.00]*	71.00 [58.10, 77.10]	72.00 [57.00, 76.50]	58.00 [49.00, 64.00]*	** < 0.001**
Ethnicity = European descent (%)	3556 (94.5)	47 (94.0)	67 (89.3)	77 (81.9)*	41 (83.7)*	19 (90.5)	33 (76.7)*	43 (75.4)*	** < 0.001**
BMI (median [IQR])	27.45 [24.46, 31.03]	28.86 [25.06, 33.29]	27.27 [24.37, 30.86]	26.52 [23.71, 31.56]	28.40 [25.88, 31.13]	25.57 [23.30, 30.27]	26.51 [24.02, 31.31]	26.37 [24.59, 30.85]	0.826
Obese = Yes (%)	1075 (40.8)	23 (65.7)	23 (44.2)	27 (42.9)	17 (44.7)	6 (46.2)	15 (44.1)	17 (34.0)	0.153
Treatment period = onset after initiating corticosteroids as standard care (%)	1649 (43.8)	22 (44.0)	37 (49.3)	64 (68.1)*	27 (55.1)	8 (38.1)	34 (79.1)*	39 (68.4)*	** < 0.001**
**Comorbidities**									
Hypertension (%)	1488 (40.0)	39 (78.0)*	55 (73.3)*	71 (77.2)*	42 (85.7)*	18 (85.7)*	35 (81.4)*	53 (93.0)*	** < 0.001**
Diabetes (%)	912 (24.7)	30 (61.2)*	37 (49.3)*	51 (54.8)*	33 (67.3)*	13 (61.9)*	24 (55.8)*	27 (47.4)*	** < 0.001**
Chronic pulmonary disease (%)	638 (17.2)	15 (30.0)	12 (16.0)	25 (26.6)	10 (20.4)	8 (38.1)	10 (23.3)	9 (15.8)	**0.01**
Chronic cardiac disease (%)	943 (25.4)	21 (42.0)*	43 (57.3)*	60 (65.2)*	28 (58.3)*	11 (52.4)*	26 (60.5)*	23 (40.4)*	** < 0.001**
Malignancy (%)	232 (6.3)	5 (10.0)	7 (9.3)	19 (20.4)*	4 (8.2)	2 (9.5)	8 (18.6)	5 (8.9)	** < 0.001**
Chronic neurological disease (%)	415 (11.2)	13 (26.0)*	15 (20.0)*	23 (24.5)*	18 (36.7)*	2 (9.5)	12 (27.9)*	8 (14.0)	** < 0.001**
Immune suppressive medication use (%)	222 (5.9)	2 (4.0)	9 (12.0)	9 (9.6)	4 (8.2)	2 (9.5)	2 (4.7)	55 (96.5)*	** < 0.001**
Erythropoietin-Stimulating Agents = 1 (%)	20 (0.5)	1 (2.0)	0 (0.0)	1 (1.1)	1 (2.0)	6 (28.6)*	11 (25.6)*	3 (5.3)*	** < 0.001**
Amount of comorbidities (median [IQR])	2.00 [1.00, 3.00]	3.00 [3.00, 5.00]*	3.00 [2.00, 4.00]*	3.00 [3.00, 4.00]*	4.00 [3.00, 4.00]*	3.00 [3.00, 4.00]*	3.00 [3.00, 4.50]*	3.00 [2.00, 4.00]*	** < 0.001**
MEWS at admission (median [IQR])	3.00 [1.00, 4.00]	3.00 [1.00, 4.00]	3.00 [2.00, 4.00]	2.00 [1.00, 4.00]	2.00 [1.00, 3.00]	2.00 [1.00, 3.00]	2.00 [1.00, 4.00]	3.00 [2.00, 4.00]	0.295
qSOFA at admission (median [IQR])	1.00 [0.00, 1.00]	1.00 [0.00, 1.00]	1.00 [0.00, 1.00]	1.00 [0.00, 1.00]	1.00 [0.00, 1.00]	0.00 [0.00, 1.00]	1.00 [0.00, 1.00]	1.00 [0.00, 1.00]	0.127
**Outcomes**									
Length of hospitalization (median [IQR])	6.00 [3.00, 12.00]	6.00 [3.00, 13.75]	8.00 [3.00, 13.00]	7.00 [3.00, 11.00]	6.00 [4.50, 9.50]	4.50 [4.00, 10.25]	9.00 [4.00, 14.50]	7.50 [3.00, 12.25]	0.188
ICU admission (%)	812 (22.2)	12 (24.5)	20 (26.7)	16 (17.0)	6 (12.5)	4 (19.0)	7 (16.7)	15 (26.3)	0.484
Invasive ventilation (%)	618 (16.9)	11 (22.9)	12 (16.2)	8 (8.5)	6 (12.5)	2 (10.0)	5 (12.2)	11 (19.3)	0.319
12-week readmission (%)	129 (3.4)	4 (8.0)	2 (2.7)	4 (4.3)	4 (8.2)	1 (4.8)	2 (4.7)	9 (15.8)*	** < 0.001**
12-week death (%)	710 (18.9)	16 (32.0)	35 (46.7)*	46 (48.9)*	24 (49.0)*	8 (38.1)	14 (32.6)	17 (29.8)	** < 0.001**
Readmission or death at 12 weeks (%)	815 (21.7)	20 (40.0)*	37 (49.3)*	49 (52.1)*	27 (55.1)*	8 (38.1)	15 (34.9)	25 (43.9)*	** < 0.001**

Significant values are in bold.

Continuous data presented as median [interquartile range], categorical in numbers (%). Continuous variables were compared using the Kruskal–Wallis test, with pairwise Wilcoxon rank tests between No CKD and the corresponding CKD group (Benjamini–Hochberg false-discovery rate adjusted) if overall significant. Significant pairs are denoted with “*”. For categorical data we used the Fisher-exact test to compare group differences , with pairwise post-hoc tests (BH adjusted) between No CKD and the corresponding CKD group if overall significant. CKD stage 2 = eGFR 60–89 ml/min/1.73 m^2^, CKD stage 3a = eGFR 45–59 ml/min/1.73 m^2^, CKD stage 3b = eGFR 30–44 ml/min/1.73 m^2^, CKD stage 4 = eGFR 15–29 ml/min/1.73 m^2^, CKD stage 5 = eGFR < 15 ml/min/1.73 m^2^, IQR = interquartile range, BMI = Body Mass Index in kg/m^2^, *MEWS* Modified Early Warning Score, *qSOFA* Quick Sequential Organ Failure Assessment, *ICU* Intensive care unit admission. *P* is considered significant at *P* ≤ 0.05.

CKD stage 2 = eGFR 60–89 ml/min/1.73 m^2^, CKD stage 3a = eGFR 45–59 ml/min/1.73 m^2^, CKD stage 3b = eGFR 30–44 ml/min/1.73 m^2^, CKD stage 4 = eGFR 15–29 ml/min/1.73 m^2^, CKD stage 5 = eGFR < 15 ml/min/1.73 m^2^.* CKD* chronic kidney disease,* BMI* body mass index in kg/m^2^.

**Table 2:** Admission vital signs and lab values in patients hospitalized for COVID-19 among chronic kidney disease groups.

	No CKD	CKD stage 2	CKD stage 3a	CKD stage 3b	CKD stage 4	CKD stage 5	Dialysis	Kidney transplantation	p value
	n = 3762	n = 50	n = 75	n = 94	n = 49	n = 21	n = 43	n = 57	
**Vital signs at admission**									
Heart rate (median [IQR])	90.00 [80.00, 102.00]	91.00 [80.25, 99.00]	90.00 [72.50, 100.00]	85.00 [70.00, 99.00]	80.00 [70.50, 94.00]	85.00 [70.50, 93.00]	86.50 [70.75, 102.75]	91.00 [82.00, 102.50]	**0.001**
Respiratory rate (median [IQR])	22.00 [18.00, 28.00]	22.00 [20.00, 28.00]	23.00 [18.00, 29.00]	22.50 [19.00, 26.50]	22.50 [17.00, 27.25]	18.00 [16.00, 23.75]	20.00 [16.00, 26.75]	24.00 [18.00, 27.50]	**0.23**
Systolic blood pressure (median [IQR])	132.00 [120.00, 148.00]	127.00 [119.00, 139.00]	132.00 [120.25, 146.75]	132.00 [119.00, 147.00]	129.00 [120.00, 147.00]	146.00 [120.75, 170.75]	135.00 [116.00, 151.00]	133.00 [114.00, 142.00]	0.148
Diastolic blood pressure (median [IQR])	79.00 [70.00, 87.00]	76.00 [70.00, 86.00]	78.00 [68.25, 84.75]	75.00 [66.00, 86.00]	66.00 [60.00, 79.00]*	76.50 [66.75, 83.25]	75.00 [65.00, 81.00]	75.00 [65.00, 87.50]	** < 0.001**
**Lab values at admission**									
Hemoglobin, mmol/L (median [IQR])	8.40 [7.70, 9.10]	7.80 [7.00, 8.50]*	7.80 [7.00, 8.65]*	7.70 [6.70, 8.50]*	6.95 [6.40, 7.90]*	6.80 [6.20, 7.50]*	6.70 [6.30, 7.40]*	7.40 [6.60, 8.60]*	** < 0.001**
White blood cell count, × 10^9^/L (median [IQR])	6.70 [5.10, 9.15]	6.50 [5.03, 8.70]	7.00 [5.15, 9.50]	6.20 [5.10, 8.10]	6.60 [5.23, 8.30]	8.10 [5.60, 10.30]	6.35 [4.73, 8.80]	6.60 [5.30, 9.30]	0.664
Lymphocyte count, × 10^9^/L (median [IQR])	0.90 [0.63, 1.24]	1.00 [0.58, 1.20]	0.80 [0.60, 1.20]	0.78 [0.50, 1.02]	0.76 [0.54, 1.05]	0.70 [0.50, 1.25]	0.72 [0.55, 1.12]	0.80 [0.46, 1.10]	**0.034**
Neutrophil count, × 10^9^/L (median [IQR])	5.00 [3.60, 7.12]	4.70 [3.92, 6.43]	5.48 [3.99, 7.62]	4.69 [3.50, 6.20]	5.52 [3.94, 7.20]	7.66 [4.64, 9.18]	3.94 [2.98, 7.14]	5.33 [3.98, 6.60]	0.248
Platelets count, × 10^9^/L (median [IQR])	214.00 [166.00, 276.00]	194.00 [153.00, 232.50]	199.00 [150.00, 258.00]	195.00 [144.50, 255.00]	196.00 [147.00, 262.00]	235.00 [185.00, 269.00]	181.00 [155.00, 213.00]	207.00 [164.50, 246.50]	**0.002**
eGFR, ml/min/1.73 m^2^ (median [IQR])	90.00 [90.00, 90.00]	67.36 [62.14, 76.22]	52.41 [48.46, 56.25]	39.39 [34.92, 42.25]	23.61 [19.66, 27.04]	11.22 [7.77, 12.48]	7.31 [5.34, 9.61]	38.11 [23.43, 52.39]	** < 0.001**
CRP, mg/L (median [IQR])	79.00 [41.00, 132.60]	93.50 [52.00, 132.90]	91.00 [45.50, 136.52]	90.00 [47.50, 142.00]	88.50 [46.88, 139.00]	99.50 [46.12, 190.45]	61.90 [37.00, 137.00]	102.40 [49.10, 139.20]	0.305
Sodium, mmol/L (median [IQR])	136.00 [133.00, 138.00]	136.00 [134.00, 139.00]	136.00 [133.00, 138.00]	135.00 [131.00, 137.00]*	136.00 [134.00, 138.00]	137.00 [134.00, 140.00]	134.50 [132.00, 138.00]	134.00 [130.00, 137.00]*	**0.001**
Potassium, mmol/L (median [IQR])	3.90 [3.60, 4.20]	4.20 [3.90, 4.50]*	4.37 [3.90, 4.80]*	4.40 [4.00, 4.80]*	4.20 [3.70, 4.70]	4.50 [4.40, 5.20]*	4.60 [4.30, 5.10]*	4.40 [4.00, 4.70]*	** < 0.001**
Calcium, mmol/L (median [IQR])	2.19 [2.10, 2.27]	2.16 [2.13, 2.22]	2.15 [2.07, 2.26]	2.20 [2.16, 2.25]	2.04 [1.96, 2.18]	2.17 [2.13, 2.19]	2.22 [2.07, 2.39]	2.24 [1.96, 2.40]	0.17
Albumine, g/L (median [IQR])	35.60 [32.00, 39.00]	36.00 [30.00, 39.00]	33.15 [30.75, 36.00]	30.40 [28.00, 35.00]*	29.00 [27.40, 34.75]*	35.50 [33.12, 37.70]	32.10 [28.10, 37.00]	35.00 [31.00, 39.00]	** < 0.001**
ALAT, U/L (median [IQR])	33.00 [22.00, 51.00]	30.00 [19.00, 49.00]	30.00 [17.75, 45.75]	27.00 [19.75, 48.00]	24.00 [20.00, 31.00]*	20.50 [17.25, 36.25]	19.00 [13.75, 28.75]*	24.00 [14.50, 33.50]*	** < 0.001**
ASAT, U/L (median [IQR])	45.00 [32.00, 66.00]	47.50 [33.50, 63.25]	50.00 [31.00, 78.00]	46.00 [36.00, 65.00]	45.00 [33.00, 58.00]	37.50 [24.00, 57.50]	37.00 [20.50, 54.50]	36.00 [28.00, 45.00]	0.061
CK, U/L (median [IQR])	126.00 [67.00, 284.00]	110.50 [55.50, 413.25]	180.00 [87.00, 284.00]	177.50 [106.50, 270.25]	193.50 [74.00, 780.75]	220.00 [126.75, 256.25]	60.00 [34.00, 475.50]	82.00 [49.50, 127.00]	0.108
LDH, U/L (median [IQR])	336.00 [260.00, 447.00]	321.00 [250.00, 490.50]	340.00 [255.00, 425.00]	342.00 [296.00, 451.00]	366.00 [280.00, 439.00]	351.00 [319.25, 455.75]	279.00 [240.50, 386.50]	308.50 [242.75, 393.00]	0.624
pH (median [IQR])	7.46 [7.43, 7.49]	7.43 [7.38, 7.45]*	7.44 [7.39, 7.46]*	7.44 [7.40, 7.46]*	7.42 [7.36, 7.44]*	7.41 [7.39, 7.45]	7.41 [7.33, 7.48]*	7.38 [7.35, 7.42]*	** < 0.001**

Significant values are in bold.

Continuous data presented as median [interquartile range], categorical in numbers (%). Continuous variables were compared using the Kruskal–Wallis test, with pairwise Wilcoxon rank tests between no CKD and the corresponding CKD group (Benjamini–Hochberg false-discovery rate adjusted) if overall significant. Significant pairs are denoted with “*” For categorical data we used the Fisher exact test to compare group differences, with pairwise posthoc tests (BH adjusted) between No CKD and the corresponding CKD group if overall significant. All pairs differed significantly in eGFR, not denoted with capitals for readability.

CKD stage 2 = eGFR 60–89 ml/min/1.73 m^2^, CKD stage 3a = eGFR 45–59 ml/min/1.73 m^2^, CKD stage 3b = eGFR 30–44 ml/min/1.73 m^2^, CKD stage 4 = eGFR 15–29 ml/min/1.73 m^2^, CKD stage 5 = eGFR < 15 ml/min/1.73 m^2^. *IQR* interquartile range, *eGFR* estimated glomerular filtration rate, *CRP* C-reactive protein, *ALAT* alanine aminotransferase, *ASAT* aspartate transaminase, *CK* creatine kinase, *LDH* lactic acid dehydrogenase.

There were multiple significant different vital signs and laboratory values at admission (Table 2). Admission diastolic blood pressure was lower in CKD stage 4 compared to no-CKD, while other vital parameters were not significantly different when comparing CKD groups to No CKD in post-hoc analyses. Hemoglobin levels were significantly lower in all CKD patients compared to no-CKD. Albumin was significantly lower in patients with CKD stage 3b and stage 4. The lymphocyte count and platelet count showed no significant difference between groups in post-hoc analyses. There were several other laboratory differences; a significant decrease of plasma sodium, increase of plasma potassium, lower plasma ALAT and lower pH in some of the CKD groups (Table 2). Most of these mean values were, however, within the normal range.

### Mortality and readmission rates

**Figure 1:**
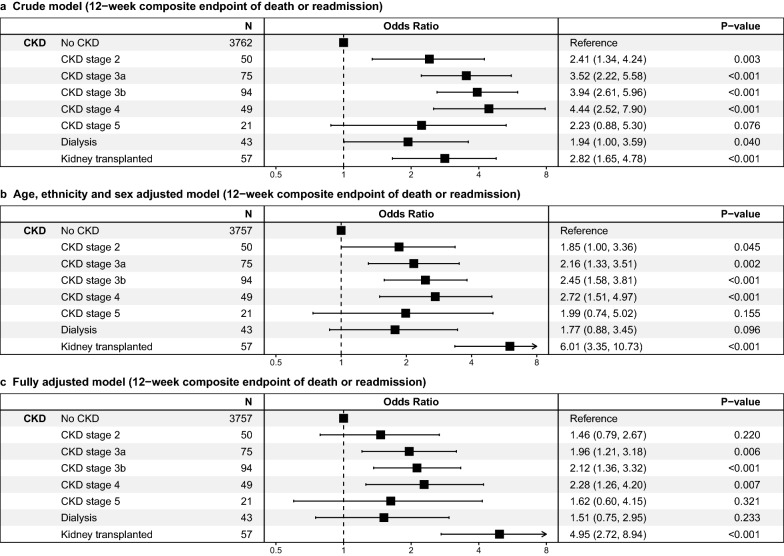
(**a**–**c**) Unadjusted; age, sex and ethnicity adjusted; and fully adjusted 12-week mortality and readmission odds ratios in patients hospitalized for COVID-19 among CKD groups compared to “no-CKD” with corresponding *P* values. CKD stage 2 = eGFR 60–79 ml/min/1.73 m^2^, CKD stage 3a = eGFR 45–59 ml/min/1.73 m^2^, CKD stage 3b = eGFR 30–44 ml/min/1.73 m^2^, CKD stage 4 = eGFR 15–29 ml/min/1.73 m^2^, CKD stage 5 = eGFR < 15 ml/min/1.73 m^2^, CKD = chronic kidney disease. *P* is considered significant at *P* ≤ 0.05.

A total of 996 patients (24.0%) died or were readmitted after 12 weeks of follow-up. In the unadjusted model, all CKD stages, except CKD stage 5, were associated with significantly higher mortality and readmission rate compared to no-CKD patients (Fig. 1a–c). After adjusting for all confounding covariables, in patients with CKD stage 3a, stage 3b, stage 4 and patients with KTX, odds ratios of death and readmission were significantly different compared to patients without CKD and ranged from 1.96 to 8.94. All CKD patients combined showed a significantly increased unadjusted and adjusted mortality and readmission rate (Supplementary Fig. 2a–c). A subgroup analyses comparing only CKD groups indicated an increased mortality and readmission rate in patients with KTX in the adjusted model. (Supplementary Fig. 3a–c).

### Complications

We observed differences in the rate of congestive heart failure, anemia requiring transfusion and stroke in various stages of CKD compared to patients without CKD. Also, the self–reported incidence of cognitive decline was higher in patients with CKD stages 2 and 3b (Table 3). All observed differences in complications were not significant in the post-hoc analyses; complications unlikely explained the higher mortality and readmission rate among CKD stages (Table 3).

**Table 3:** Complications among chronic kidney disease groups during hospitalization for COVID-19.

	No CKD	CKD stage 2	CKD stage 3a	CKD stage 3b	CKD stage 4	CKD stage 5	Dialysis	Kidney transplantation	p value
	n = 3762	n = 50	n = 75	n = 94	n = 49	n = 21	n = 43	n = 57	
**Infectious**									
Bacteremia (%)	190 (5.5)	7 (15.6)	5 (7.2)	2 (2.3)	2 (4.5)	2 (10.5)	3 (7.7)	5 (8.9)	0.076
Bacterial pneumonia (%)	428 (12.5)	6 (13.6)	7 (10.1)	9 (10.5)	4 (9.3)	7 (36.8)	6 (14.6)	7 (12.7)	0.117
Aspergillosis pneumonia (%)	81 (2.5)	3 (7.9)	4 (6.7)	1 (1.2)	2 (4.8)	0 (0.0)	1 (2.4)	3 (5.5)	0.107
Endocarditis/myocarditis/pericarditis (%)	16 (0.5)	0 (0.0)	0 (0.0)	0 (0.0)	1 (2.3)	0 (0.0)	0 (0.0)	1 (1.8)	0.501
**Pulmonary**									
Acute respiratory distress syndrome (%)	507 (14.8)	5 (11.1)	10 (14.5)	5 (5.7)	7 (15.9)	2 (10.5)	3 (7.3)	3 (5.4)	0.107
Pneumothorax (%)	54 (1.6)	0 (0.0)	0 (0.0)	0 (0.0)	0 (0.0)	0 (0.0)	1 (2.4)	0 (0.0)	0.623
**Cardiac**									
Cardiac arrhythmia (%)	266 (7.7)	6 (13.6)	4 (5.8)	10 (11.5)	1 (2.3)	0 (0.0)	1 (2.4)	4 (7.1)	0.228
Cardiac ischaemia (%)	57 (1.7)	2 (4.4)	2 (2.9)	2 (2.3)	0 (0.0)	1 (5.6)	1 (2.4)	1 (1.8)	0.633
Cardiac arrest (%)	812 (22.2)	12 (24.5)	20 (26.7)	16 (17.0)	6 (12.5)	4 (19.0)	7 (16.7)	15 (26.3)	0.484
Congestive heart failure (%)	94 (2.7)	3 (6.7)	1 (1.4)	5 (5.7)	5 (11.1)	2 (10.5)	2 (4.9)	1 (1.8)	**0.004**
**General**									
Physical decline (%)	910 (27.3)	14 (32.6)	27 (39.1)	34 (40.0)	13 (31.7)	9 (47.4)	11 (26.8)	14 (25.0)	**0.029**
Cognitive decline (%)	232 (7.0)	8 (18.6)	8 (12.1)	18 (21.2)	7 (17.1)	1 (5.3)	7 (17.1)	4 (7.3)	** < 0.001**
Delirium (%)	413 (12.1)	10 (22.7)	14 (20.9)	15 (17.6)	8 (19.5)	4 (20.0)	8 (19.5)	6 (10.7)	**0.03**
Anemia requiring transfusion (%)	166 (4.8)	3 (6.7)	6 (8.8)	4 (4.6)	6 (13.3)	4 (22.2)	5 (12.2)	4 (7.1)	**0.001**
Liver failure (%)	41 (1.2)	0 (0.0)	1 (1.4)	0 (0.0)	1 (2.3)	0 (0.0)	1 (2.4)	2 (3.6)	0.608
**Coagulation disorders**									
Deep venous thrombosis (%)	56 (1.5)	0 (0.0)	0 (0.0)	1 (1.1)	0 (0.0)	0 (0.0)	1 (2.3)	3 (5.3)	0.259
Pulmonary embolism (%)	147 (3.9)	1 (2.0)	3 (4.0)	1 (1.1)	1 (2.0)	1 (4.8)	1 (2.3)	3 (5.3)	0.83
Stroke (%)	64 (1.7)	1 (2.0)	6 (8.0)	3 (3.2)	0 (0.0)	0 (0.0)	0 (0.0)	0 (0.0)	**0.004**
Superficial thrombophlebitis (%)	20 (0.5)	0 (0.0)	0 (0.0)	0 (0.0)	0 (0.0)	0 (0.0)	0 (0.0)	0 (0.0)	0.955

Significant values are in bold.

Continuous data presented as median [interquartile range], categorical in numbers (%). Continuous variables were compared using the Kruskal–Wallis test, with pairwise Wilcoxon rank tests between No CKD and the corresponding CKD group (Benjamini–Hochberg false-discovery rate adjusted) if overall significant. Significant pairs are denoted with “*”. For categorical data we used the Fisher exact test to compare group differences, with pairwise posthoc tests (BH adjusted) between No CKD and the corresponding CKD group if overall significant. All pairs differed significantly in eGFR, not denoted with capitals for readability.*P* is considered significant at *P* ≤ 0.05.

CKD stage 2 = eGFR 60–89 ml/min/1.73 m^2^, CKD stage 3a = eGFR 45–59 ml/min/1.73 m^2^, CKD stage 3b = eGFR 30–44 ml/min/1.73 m^2^, CKD stage 4 = eGFR 15–29 ml/min/1.73 m^2^, CKD stage 5 = eGFR < 15 ml/min/1.73 m^2^. *IQR* interquartile range, *ARDS* acute respiratory distress syndrome, *DVT* deep venous thrombosis.

## Discussion

Our multicenter cohort of admitted COVID-19 patients demonstrates overall increased mortality and readmission rates in patients with CKD. We show a strong unadjusted increased clinical mortality and readmission rate in patients with any form of CKD compared to patients without CKD, except for CKD stage 5. After full adjustments, in patients with CKD stages 3a, 3b, 4 and in KTX patients, the odds ratios remain significantly increased compared to patients without CKD. Notably, the odd ratios within CKD groups did not indicate any association with CKD stage severity. We could not identify the main complication responsible for the higher mortality and readmission rate. We also could not identify clear patterns in vital signs or laboratory values at admission that explain increased mortality and readmission rate across CKD groups in our cohort, aside from lower diastolic blood pressure (CKD stage 4) and significant differences in hemoglobin and albumin—the latter being presumably directly related to CKD stage rather than COVID-19. The lack of an apparent factor that explains worse 12-week outcomes underscores that CKD as such is a COVID-19 risk factor.

However, some distinctions can be made, as we also analyzed mortality and readmission rates among CKD patients with dialysis and KTX. Our findings are in concordance with the ERACODA collaborative study, where a higher mortality rate was found among KTX and dialysis patients than data from the general population^4^. However, in contrast to KTX patients from our cohort, dialysis patients (predominantly hemodialysis) do not appear to have significantly higher mortality and readmission rates after adjusting for known covariates. The significantly higher mortality after correction demonstrates that mortality and readmission rates in the KTX group are not dependent on known COVID-19 risk factors such as older age, male sex and ethnicity^14,15^. This is in contrast with our findings in dialysis patients in whom the worse 12-week outcomes to some extent seem to be explained by higher age and more prevalent comorbidities associated with worse COVID-19 outcomes in non-renal patients.

It has been suggested that COVID-19 associated mortality in patients with KTX is due to the immunosuppressive condition of patients, which leads to an inability to clear the SARS-CoV-2 infection^16^. Various studies have also shown a limited yield of SARS-CoV-2 vaccines in patients with a solid transplantation^16–18^. This study again emphasizes the present-day increased risk of mortality in patients with KTX. Besides KTX patients, usage of immune suppressive medication in other CKD groups was not significantly different from the no-CKD group in our cohort.

There are some potential drawbacks associated with our study. First, although we can draw conclusions on the influence of CKD on COVID-19 outcomes, the influence of CKD severity was studied based on eGFR staging only. We were not able to include albuminuria stage, which could affect CKD severity and its association with outcomes. Furthermore, due to requiring pre-admission creatinine values, we have excluded 247 CKD patients. However, we believe this selection provides a more accurate representation of mortality within CKD groups. We also did not study the impact of the primary diagnosis causing CKD on the course of COVID-19 because these data were not completely available. At last, there are differences in baseline characteristics and in designated COVID-19 treatment wave, which might have resulted in different treatment regimens among groups. Although we adjusted for potential confounders, there remains a possibility of an overestimation or underestimation of results. However, we do believe this only makes our results more applicable to general clinical conditions in hospitals.

## Conclusion

Our study demonstrates a clinically significant increased mortality and readmission rate in patients with a history of CKD. While previous studies mainly highlighted increased mortality among dialysis and KTX patients, we also demonstrate a clear increased 12-week mortality and readmission rate of SARS-CoV-2 infected patients in nearly all CKD groups. Besides justified concerns for KTX patients, clinicians should also be aware of more severe COVID-19 outcomes and increased vulnerability in CKD patients**.**

## Supplementary Information

Supplementary Information.

## Data Availability

The original study protocol and data dictionary will be made available to researchers upon request. Researchers willing to access the de-identified participant dataset should send a request to l.vogt@amsterdamumc.nl. Requests for data will be evaluated, and access will depend on the informed consent and permission of legal research support of Amsterdam UMC.
