# Supplementary material for: Mortality and readmission rates among hospitalized COVID-19 patients with varying stages of chronic kidney disease: a multicenter retrospective cohort
Source: Sci Rep. 2022 Feb 10;12:2258. doi: 10.1038/s41598-022-06276-7 (PMC8831646; doi:10.1038/s41598-022-06276-7)
Supplement: Supplementary file 1 — Supplementary Information. [file 41598_2022_6276_MOESM1_ESM.docx]

**Supplementary material section**

**Figure legends supplementary file.**

**Supplemental Figure S1.** Flowchart of inclusion

**Supplemental Figure S2a-c.** Unadjusted; age, sex and ethnicity adjusted; and fully adjusted 12-week mortality and readmission odds ratios among CKD stages combined compared to “no-CKD” with corresponding *P* values.

CKD = chronic kidney disease. *P* is considered significant at P ≤ 0.05.

**Supplemental Figure S3a-c.** Unadjusted, age, sex and ethnicity adjusted; and fully adjusted 12-week mortality and readmission odds ratios among patients with CKD with corresponding *P* values

CKD stage 2 = eGFR 60-79 ml/min/1,73 m^2^, CKD stage 3a = eGFR 45-59 ml/min/1,73 m^2^, CKD stage 3b = eGFR 30-44 ml/min/1,73 m^2^, CKD stage 4 = eGFR 15-29 ml/min/1,73 m^2^, CKD stage 5 = eGFR <15 ml/min/1,73 m^2^, CKD = chronic kidney disease. *P* is considered significant at P ≤ 0.05.

**Supplemental figure S1.**


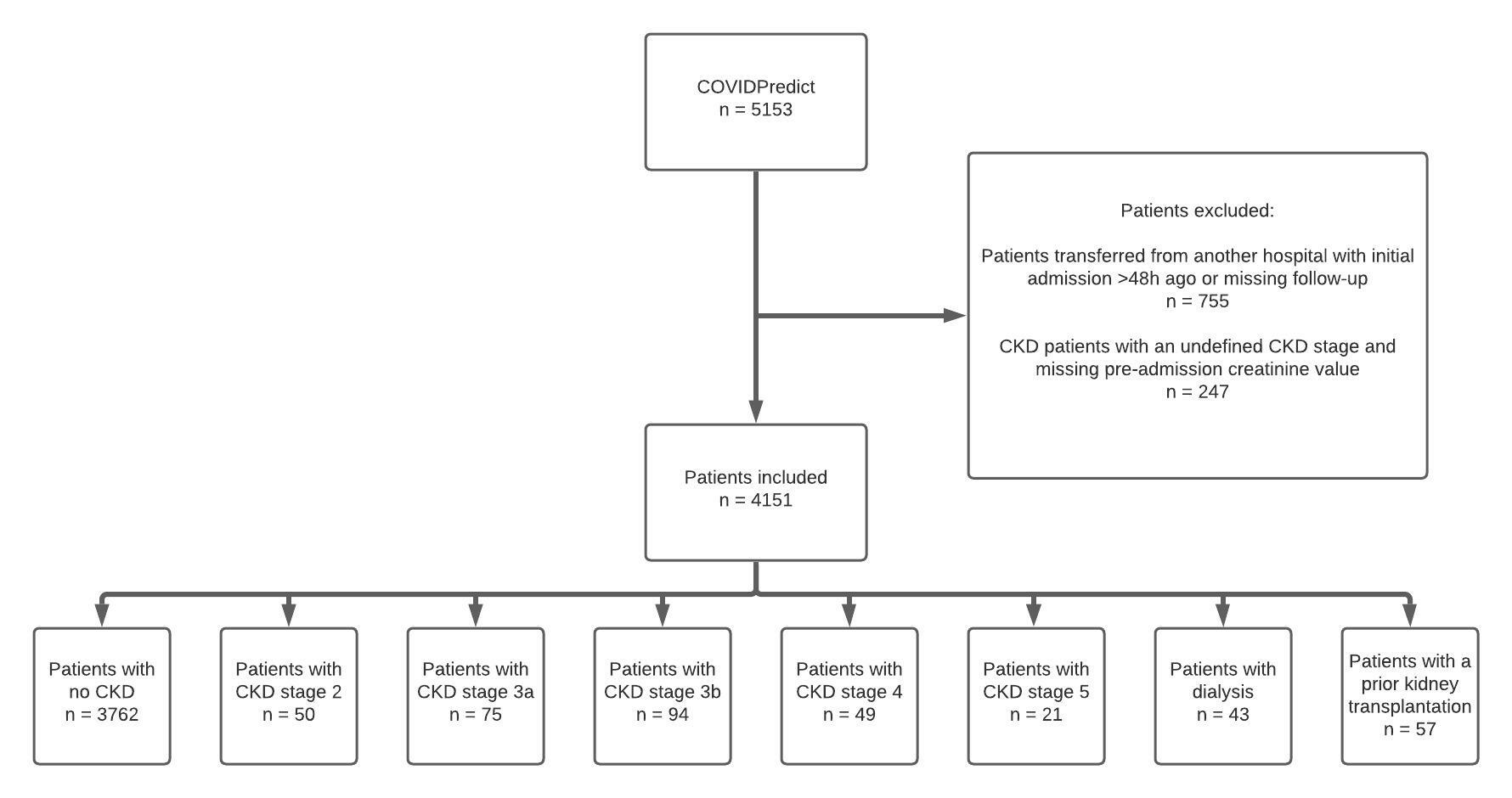


**Supplemental figure S2a-c.**

**
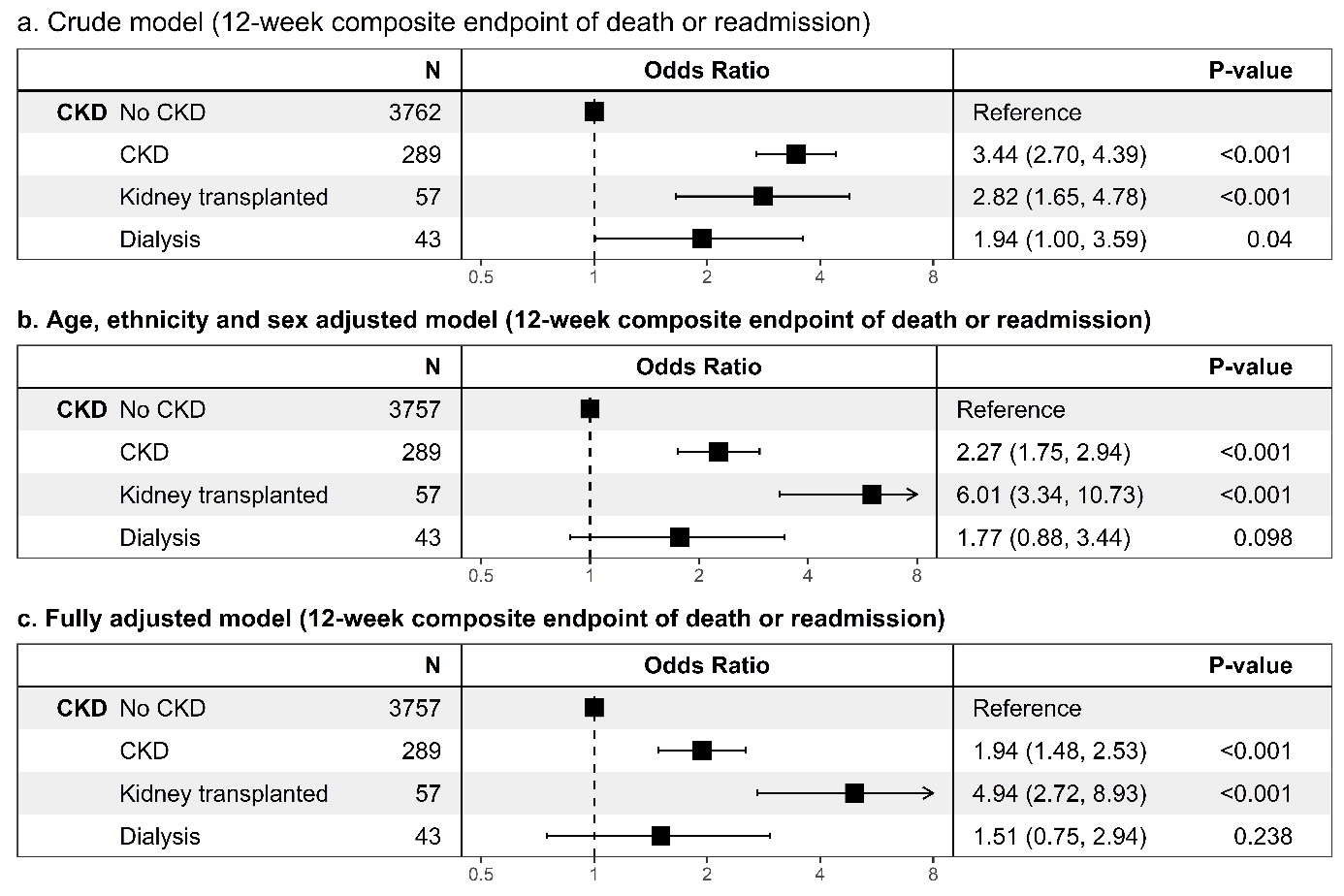
**

**Supplemental figure S3a-c.**

**
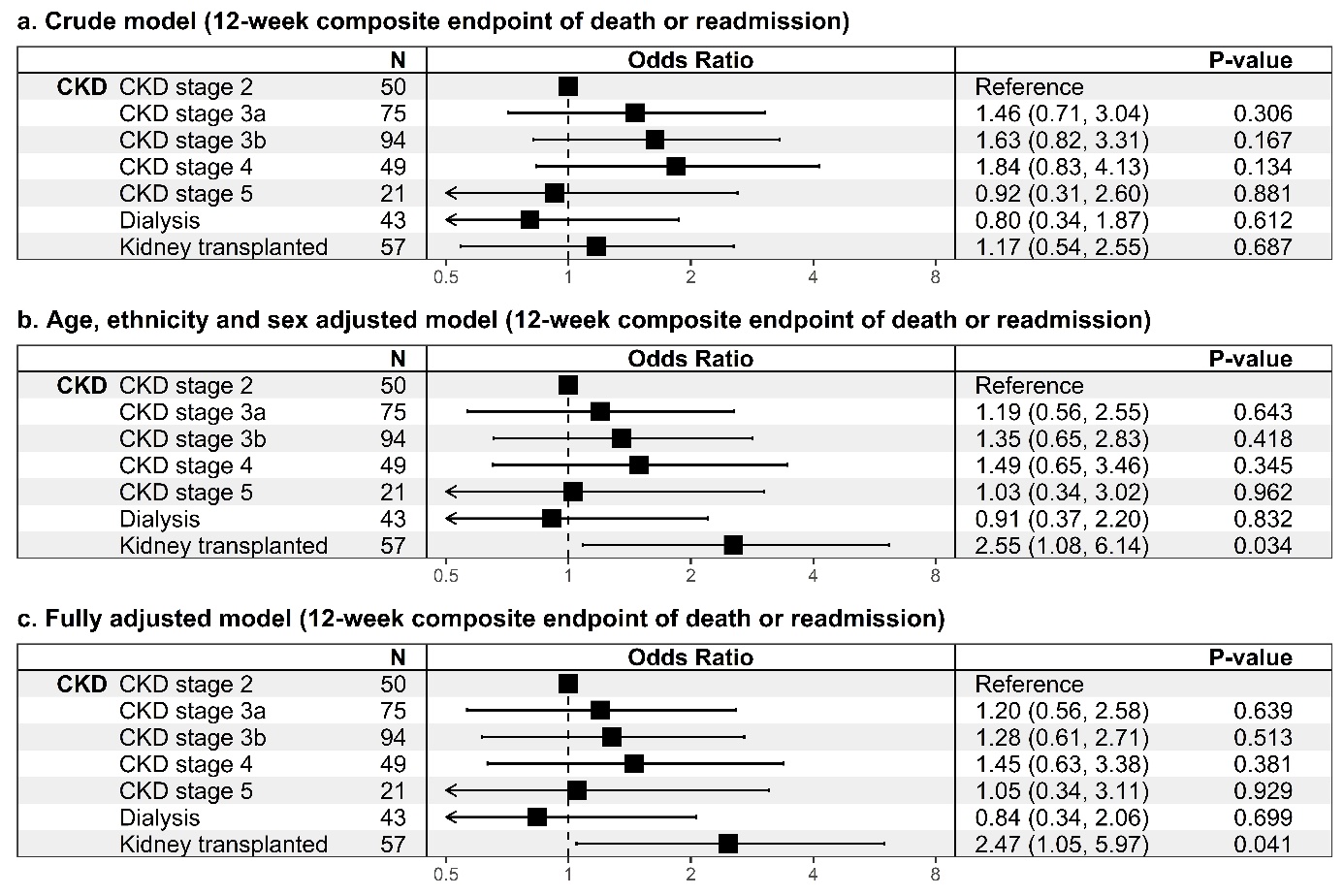
**
